# Supplementary figures and images for: The impact of metastatic sites in advanced pancreatic adenocarcinoma, systematic review and meta-analysis of prospective randomized studies
Source: PLoS One. 2020 Mar 4;15(3):e0230060. doi: 10.1371/journal.pone.0230060 (PMC7055903; doi:10.1371/journal.pone.0230060)

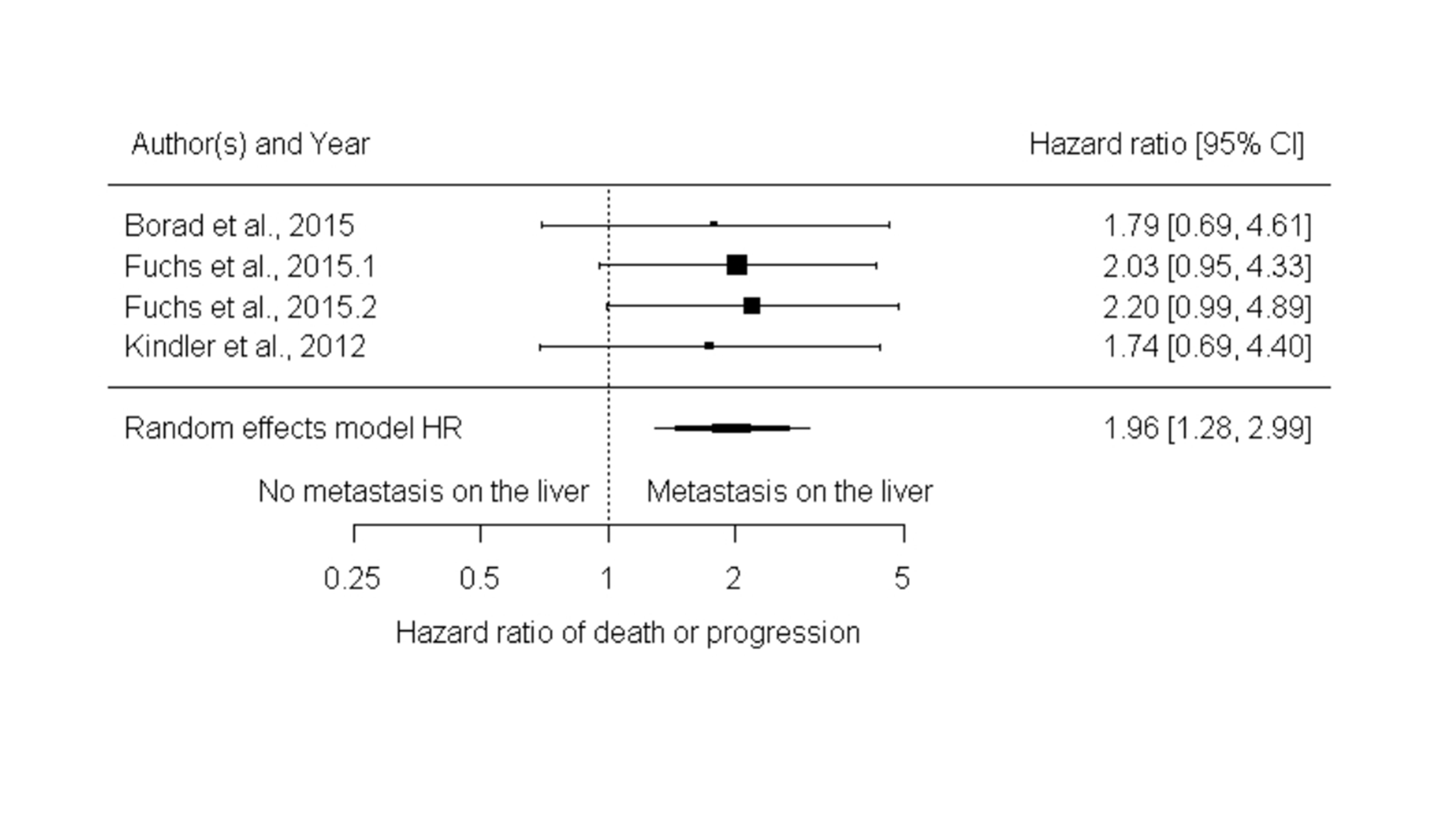

Supplement: S1 Fig — (TIF) [file pone.0230060.s001.tif]
